# Weissella confusa Causing Vancomycin-Resistant Septicemia Infection in a Pediatric Patient: A Case Report From a University Teaching Hospital in North India

**DOI:** 10.7759/cureus.38292

**Published:** 2023-04-29

**Authors:** Amber Azim, Nishtha Singh, Vimala Venkatesh, Sheetal Verma, Avinash Agarwal

**Affiliations:** 1 Microbiology, King George's Medical University, Lucknow, IND; 2 Critical Care Medicine, King George's Medical University, Lucknow, IND

**Keywords:** vancomycin resistant, polymicrobial infection, review, case report, bacteriemia

## Abstract

*Weissella confusa *is a Gram-positive coccus usually found in the microbiota of humans and the environment. Different studies quote that it has caused infections in humans under unfavourable conditions. A case report causing septicemia* *in an 11-year-old male patient diagnosed with acute pancreatitis and having acute respiratory distress syndrome (ARDS) is presented. The patient was successfully treated with ceftazidime and a piperacillin-tazobactam combination after confirmation of bacteria by matrix-assisted laser desorption and ionisation-time of flight (MALDI-TOF-MS) and antimicrobial sensitivity testing (AST) performed as per the latest Clinical and Laboratory Standard Institute (CLSI) guidelines. The patient was discharged asymptomatically after drainage of fluid and was managed conservatively. Correct identification by the automated method is important for this species and also to find its mode of infection. Because of its similarities to other vancomycin-resistant cocci, isolates of this species might be difficult to identify, leading to drug resistance. A literature review in tabulated form is summarised.

## Introduction

*Weissella confusa* is a rarely isolated Gram-positive, catalase-negative, non-motile cocci that shows few similar characteristics, such as the ability to produce acid and aroma, a strong tolerance to low pH, and the ability to ferment foods like some lactic acid bacteria [1,2]. It is a facultative anaerobe that requires optimal conditions for its growth and metabolism. It has also been postulated that it shows drug resistance to vancomycin due to its empirical use without proper diagnosis [3]. It was earlier known as Lactobacillus confuses, which is generally confused with members of the Leuconostoc, Pediococcus, and Lactobacillus genera [4]. 'Weissella,' the term, was named after the German microbiologist Norbert Weiss [5]. It is found as a member of normal gut bacteria, and the gastrointestinal tract is found to be its reservoir for colonisation [3,6]. Automated identification systems are currently being practised by various laboratories to identify this species correctly. Among the 22 recognised species of Weissella, this species is found to be most frequently associated with human infections. Establishing its pathogenicity and accurately making its diagnosis by traditional phenotypic testing methods is the challenge. Bacteremia is reported to be the most frequent infection caused by this organism. It is also found to be isolated in patients with other co-infections, especially in the gastrointestinal tract [7]. Patients with endocarditis, abscess infection, osteoarthritis, and ceacal carcinoma have also reported infection with other microorganisms causing septicemia [8]. The majority of serious infections have been reported in immunocompromised patients with co-morbidities. Its isolation from polymicrobial infection has not yet been significantly studied; more literature reviews are required in this field. Here, we present an interesting case of *W. confusa* in a paediatric patient with acute pancreatitis. The main objective of this case report is accurate identification to prevent drug resistance and analysis of the root cause of infection.

## Case presentation

An 11-year-old male child was admitted to the Critical Care Medicine Unit in our hospital with chief complaints of high-grade fever, difficulty breathing, severe tachycardia, and tachypnea. The family provided a history of treatment at a private hospital for high-grade fever (102 °F), abdominal pain, distention, and recurrent episodes of vomiting 24 days ago, where investigations showed serum aspartate aminotransferase (AST) greater than 250 IU/L and serum lactate dehydrogenase (LDH) greater than 400 IU/L, and the patient was diagnosed as suffering from acute pancreatitis with severe inflammatory response syndrome (SIRS) and evolving acute respiratory distress syndrome (ARDS). He was managed conservatively there; contrast-enhanced computed tomography (CECT) performed showed extensive necrosis, following which a left-sided percutaneous drain was placed and 1500 ml of fluid was drained. The patient was started on imipenem, piperacillin, and metronidazole after temporary relief and left the hospital against medical advice. After five days of discharge, the patient was again admitted to our hospital with the complaints mentioned above. He was assessed and diagnosed as a follow-up case of acute pancreatitis with nosocomial pneumonia.

After admission, vitals were monitored regularly; on examination, blood pressure was 117/78 mmHg, heart rate was 110/min, and on auscultation, bilateral crept were heard. SpO_2_ 96%, electrolyte Na - 130, K - 3.9, Ca - 3.64, Mg - 1.71. Hb - 7.5 g/dl, platelets - 1.7 lakhs/mm^3^, procalcitonin - 2.25 ng/ml, total leucocyte count - 10,600 cells/mm^3^ (neutrophils - 60%, lymphocytes - 36%, eosinophils - 02%, monocytes - 02%, basophils - 00%), total red blood cells (RBCs) 3.27 million cells/µl. Serum phosphorous - 5.63 mg/dl, serum urea - 29.2 mg/dl, serum creatinine - 0.84 mg/dl, serum bilirubin total - 0.33 mg/ dl, serum bilirubin direct - 0.17 mg/dl, serum glutamic-oxaloacetic transaminase (SGOT) - 37.6 IU/L, serum glutamic pyruvic transaminase (SGPT) - 49.1 IU/L, serum alkaline phosphatase - 179 IU/L, prothrombin time - 14.5 sec, INR - 1.08, viral markers - non-reactive. The patient was started empirically on polymyxin, vancomycin, and other broad-spectrum antibiotics: amoxicillin-clavulanic acid, cefepime, gentamicin, levofloxacin, meropenem, piperacillin-tazobactam, and ceftazidime to give both Gram-negative and Gram-positive coverage. Ultrasonography of the abdomen showed residual fluid in the left paracolic gutter, for which percutaneous drainage (PCD) was done. After 48 hours, the patient started taking oral feeds, and his vitals were monitored regularly.

**Figure 1:**
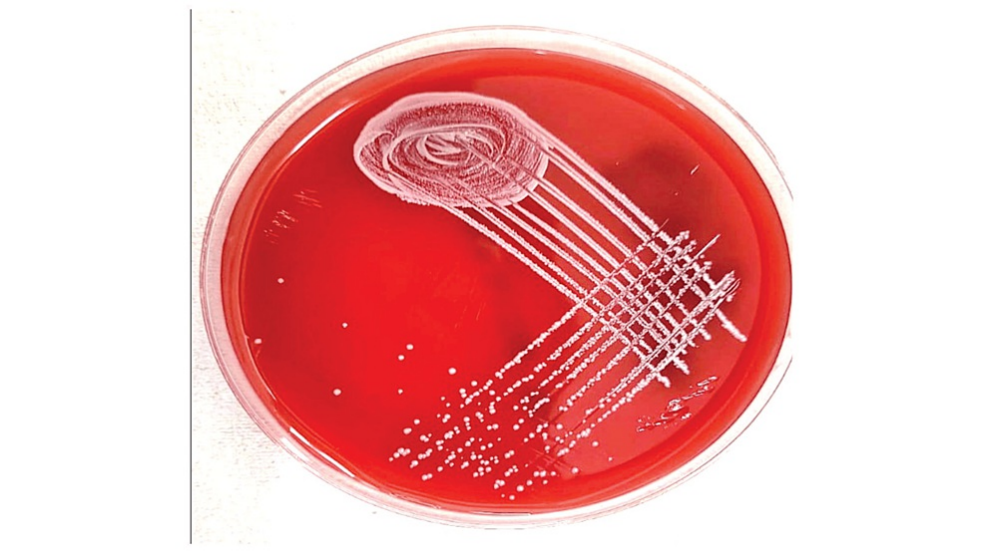
Sheep blood agar showing opaque, semi-transparent, and convex shaped colonies of Weissella confusa

Blood, urine, and drain fluid samples were sent for bacterial aerobic culture and sensitivity testing to the Department of Microbiology. Left-sided percutaneous drain fluid samples and urine on culture were sterile after 48 hours of incubation at 37 °C. A blood culture bottle was inserted in the BACTEC machine, which beeped positive, and on culture, the sheep blood agar and MacConkey agar were incubated at 37 °C for 24 hours. The colonies on sheep blood agar were white, opaque, and semi-transparent. Gram-stain showed cocci that were lenticular and spherical in shape (Figure 1).

The bacterial identification was done by MALDI-TOF (Biomeriux, France), and antibiotic susceptibility was performed by the Kirby-Bauer disc diffusion method as per the Clinical and Laboratory Standard Institute (CLSI) 2022 guidelines. The isolate was susceptible to amoxicillin-clavulanic acid, cefepime, gentamicin, levofloxacin, meropenem, piperacillin-tazobactam, and ceftazidime. It was resistant to cefoxitin, aztreonam, and vancomycin, which were stopped. Repeat blood cultures after one week also showed growth of Candida parapsilosis, which was sensitive to fluconazole, amphotericin B, itraconazole, and voriconazole, along with the re-isolation of Weisella confusa species with a susceptibility pattern similar to the previous report. The vancomycin and colistin were stopped, and the patient was started on piperacillin-tazobactam 2.5 g, ceftazidime 25 mg/kg/dose, and voriconazole 100 mg BD, which were administered for 20 days. The patient showed significant clinical improvement and was successfully discharged 30 days post-admission. On discharge, he was prescribed multivitamins, multi-minerals, and calcium supplements. Again, on evaluation after 15 days, there were no signs of progression of the disease, and all the vital parameters were normal.

## Discussion

Here, a case of infection in an 11-year-old child with vomiting and respiratory distress caused by polymicrobial infections involving *W. confuse* and *Candida parapsilosis* is described. Members of this genus are alpha-hemolytic, Gram-positive coccobacilli that usually grow in chains. They are vancomycin-resistant and catalase-negative, showing a pyrrolidinyl-β-naphthylamide (PYR) negative reaction with bile-esculin positive [4]. Analysis by MALDI-TOF is essential for the identification of bacteria from clinical specimens, as they may be missed or misidentified. This strain of these species has also been isolated from various samples like cerebrospinal fluid, infected wounds, and stool [2]. This bacteria is generally found with another organism as a co-infection, especially in individuals with suppressed immunity. Other conditions like a prolonged hospital stay, drug resistance, and altered gut flora have proven to favour its infection in immunocompetent individuals as well. Gastrointestinal problems like hepatobiliary jaundice, cholecystitis, and cholelithiasis are favourable factors for this bacteria to cause infection [8]. The drug of choice must be based on antimicrobial susceptibility testing, the patient's health profile, and the site of its isolation and infection. It is intrinsically resistant to vancomycin and therefore shows high minimum inhibitory concentrations [5]. Drug resistance is important to identify as physicians often use drugs empirically as a treatment option where culture initially reveals Gram-positive cocci. This leads to the development of vancomycin-resistant infections and false diagnoses. A case series of 10 clinical isolates was done in which they could not be correctly identified to a species level. With VITEK 2 and Phoenix commercial automated identification systems, it is possible to identify it accurately [6]. It is often found to be misidentified with viridans, streptococci, enterococci, and a few other lactic acid-fermenting bacteria [9-11]. The molecular method of 16S sequencing is the gold standard for its identification. MALDI-TOF-MS has currently been proven to be a rapid and easy tool for identification, but its application is difficult in routine microbiology laboratories [12]. This organism is also habitat of various environmental sources reported to thrive in temperatures ranging from 15 to 40 °C. Studies on treatment accuracy for septicaemia with *W. confusa* were conducted, which show the prescription of daptomycin, amoxicillin-clavulanate, or piperacillin/tazobactam as the most treatable options. Most cases of its infection are found in patients with invasive diseases or those with suppressed immunity. Generally, translocation from gut flora is the most common mode of infection, especially in immunocompromised as well as immunocompetent individuals and individuals with prolonged hospital stays showing multi-drug-resistant species [13]. Its remarkable antimicrobial and anti-inflammatory properties support its probiotic potential. With significant research backing the use of Weissella, it may be recognised as an important probiotic in the near future, having some application across industries. The literature is less focused on probiotic support and polymicrobial infection; more research is required in this field for patients health and appropriate treatment at the correct time. The main management lies in an accurate culture report for correct treatment. A table of different literature studies is given below (Table 1).

**Table 1:** Comparative analysis of Weissella confusa causing various infections with co-species in different literature

Sex (male (M), female (F); age (years)	Underlying conditions	Clinical infection	Co-infection	Treatment	Outcome
M, 25	Hepatocellular carcinoma, Liver transplant, hepatic artery thrombosis, diabetes	Bacteremia	Aeromonas hydrophila	Metronidazole and levofloxacin	Cured [14]
F, 58	Gastroesophageal adenocarcinoma	Bacteremia	*Acinetobacter baumannii*, *Candida albicans*	Cefoperazone-sulbactam Metronidazole	Cured [15]
M, 73	Hypertension, aortic intramural hematoma	Bacteremia	Chryseobacterium indologenes	Teicoplanin and piperacillin-tazobactam	Cured [16]
M, 64	Crohn’s disease with gastrointestinal strictures, central venous catheter	Bacteremia	Enterococcus faecalis	Piperacillin/tazobactam	Cured [17]
F, 12	Crohn’s disease, short bowel syndrome, intestinal failure	Bacteremia	Polymicrobial	Meropenem, metronidazole, and cefuroxime	Cured [18]
M, 11	Acute pancreatitis with acute respiratory distress syndrome	Bacteremia	Candida parapsilosis	Piperacillin-tazobactam and ceftazidime	This study

## Conclusions

Here, we describe a case report showing an unusual case of *W. confusa* in an acute pancreatitis patient. It appears to be an opportunistic bacteria that can rarely cause blood infections and warrants rapid and accurate identification to ensure prompt antibiotic therapy. Antimicrobial susceptibility testing is vital to guiding appropriate therapy in cases of severe infections. The drugs, including vancomycin, metronidazole, rifampin, teicoplanin, ceftazidime, and trimethoprim-sulfamethoxazole, should not be administered to patients with Weissella infections. More literature review and study are required for this organism to be associated with polymicrobial and bloodstream infections as hospital-acquired infections or opportunistic pathogens.

## References

[1] Collins MD, Samelis J, Metaxopoulos J, Wallbanks S (1993). Taxonomic studies on some leuconostoc-like organisms from fermented sausages: description of a new genus Weissella for the Leucon- ostoc paramesenteroides group of species. J Appl Bacteriol.

[2] Fusco V, Quero GM, Cho GS (2015). The genus Weissella: taxonomy, ecology and biotechnological potential. Front Microbiol.

[3] Fusco V, Quero GM, Stea G, Morea M, Visconti A (2011). Novel PCR-based identification of Weissella confusa using an AFLP-derived marker. Int J Food Microbiol.

[4] Kim E, Yang SM, Kim HY (2023). Weissella and the two Janus faces of the genus. Appl Microbiol Biotechnol.

[5] Salimnia H, Alangaden GJ, Bharadwaj R, Painter TM, Chandrasekar PH, Fairfax MR (2010). Weissella confusa: an unexpected cause of vancomycin-resistant Gram-positive bacteremia in immunocompromised hosts. Transpl Infect Dis.

[6] Hurt W, Savarimuthu S, Mughal N, Moore LS (2021). A rare case of Weissella confuse endocarditis. Clin Infect Pract.

[7] Medford R, Patel SN, Evans GA (2014). A confusing case - Weissella confusa prosthetic joint infection: A case report and review of the literature. Can J Infect Dis Med Microbiol.

[8] Flaherty JD, Levett PN, Dewhirst FE, Troe TE, Warren JR, Johnson S (2003). Fatal case of endocarditis due to Weissella confusa. J Clin Microbiol.

[9] Shin JH, Kim DI, Kim HR, Kim DS, Kook JK, Lee JN (2007). Severe infective endocarditis of native valves caused by Weissella confusa detected incidentally on echocardiography. J Infect.

[10] Fairfax MR, Lephart PR, Salimnia H (2014). Weissella confusa: problems with identification of an opportunistic pathogen that has been found in fermented foods and proposed as a probiotic. Front Microbiol.

[11] Ahmed S, Singh S, Singh V, Roberts KD, Zaidi A, Rodriguez-Palacios A (2022). The Weissella genus: Clinically treatable bacteria with antimicrobial/probiotic effects on inflammation and cancer. Microorganisms.

[12] Harlan NP, Kempker RR, Parekh SM, Burd EM, Kuhar DT (2011). Weissella confusa bacteremia in a liver transplant patient with hepatic artery thrombosis. Transpl Infect Dis.

[13] Kumar A, Augustine D, Sudhindran S, Kurian AM, Dinesh KR, Karim S, Philip R (2011). Weissella confusa: a rare cause of vancomycin-resistant Gram-positive bacteraemia. J Med Microbiol.

[14] Lee W, Cho SM, Kim M, Ko YG, Yong D, Lee K (2013). Weissella confusa bacteremia in an immune-competent patient with underlying intramural hematomas of the aorta. Ann Lab Med.

[15] Vasquez A, Pancholi P, Balada-Llasat JM (2015). Photo quiz: confusing bacteremia in a Crohn's disease patient. Weissella confusa. J Clin Microbiol.

[16] Spiegelhauer MR, Yusibova M, Rasmussen IK, Fuglsang KA, Thomsen K, Andersen LP (2020). A case report of polymicrobial bacteremia with Weissella confusa and comparison of previous treatment for successful recovery with a review of the literature. Access Microbiol.

[17] Olano A, Chua J, Schroeder S, Minari A, La Salvia M, Hall G (2001). Weissella confusa (basonym: Lactobacillus confusus) bacteremia: a case report. J Clin Microbiol.

[18] Riebel WJ, Washington JA (1990). Clinical and microbiologic characteristics of pediococci. J Clin Microbiol.

